# Supplementary figures and images for: SPACR Encoded by IMPG1 Is Essential for Photoreceptor Survival by Interplaying between the Interphotoreceptor Matrix and the Retinal Pigment Epithelium
Source: Genes (Basel). 2022 Aug 23;13(9):1508. doi: 10.3390/genes13091508 (PMC9498744; doi:10.3390/genes13091508)

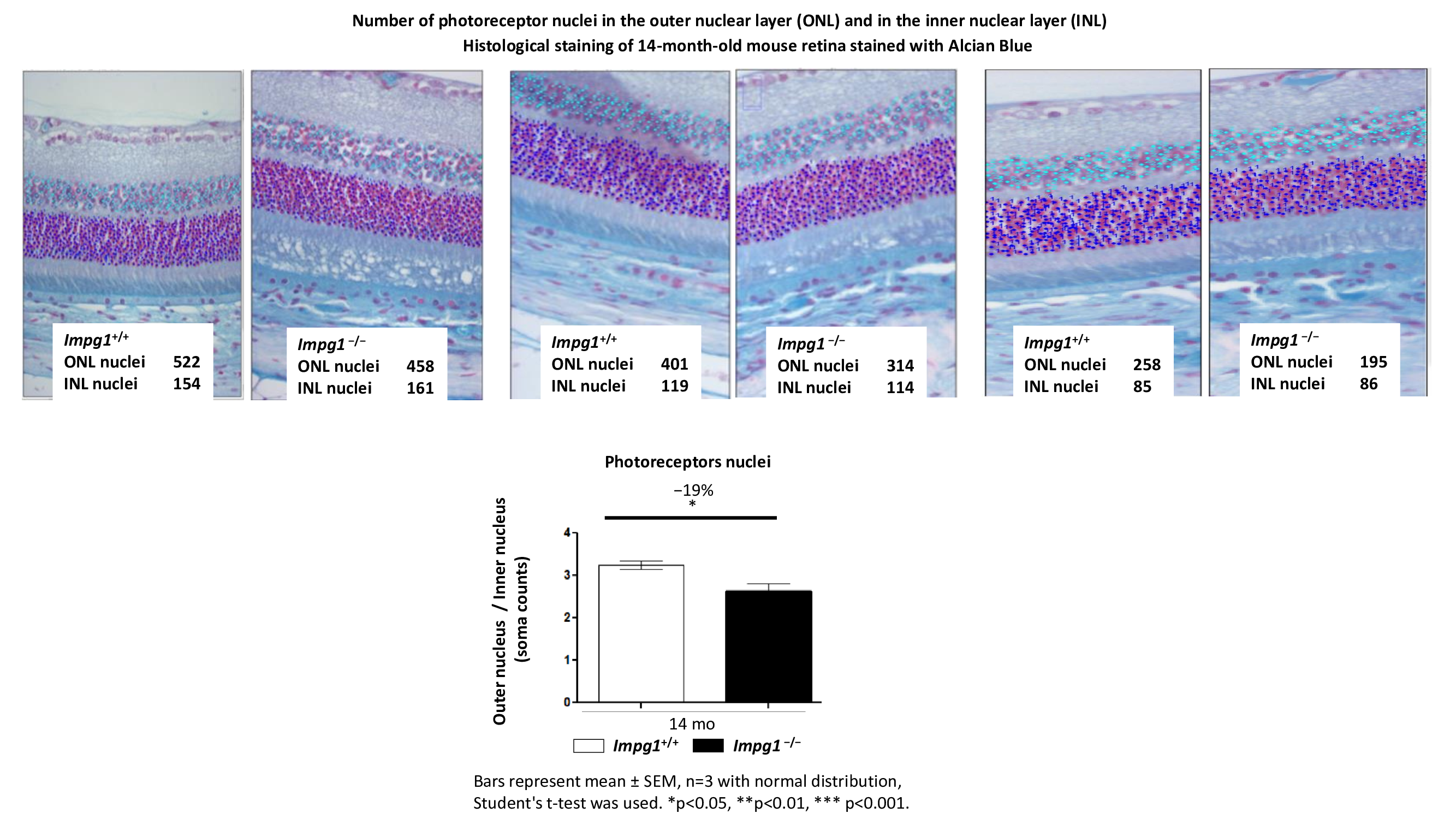

Supplement: Supplementary file 1 [file genes-13-01508-s001.zip › Supplementary_figure_S2.tiff]

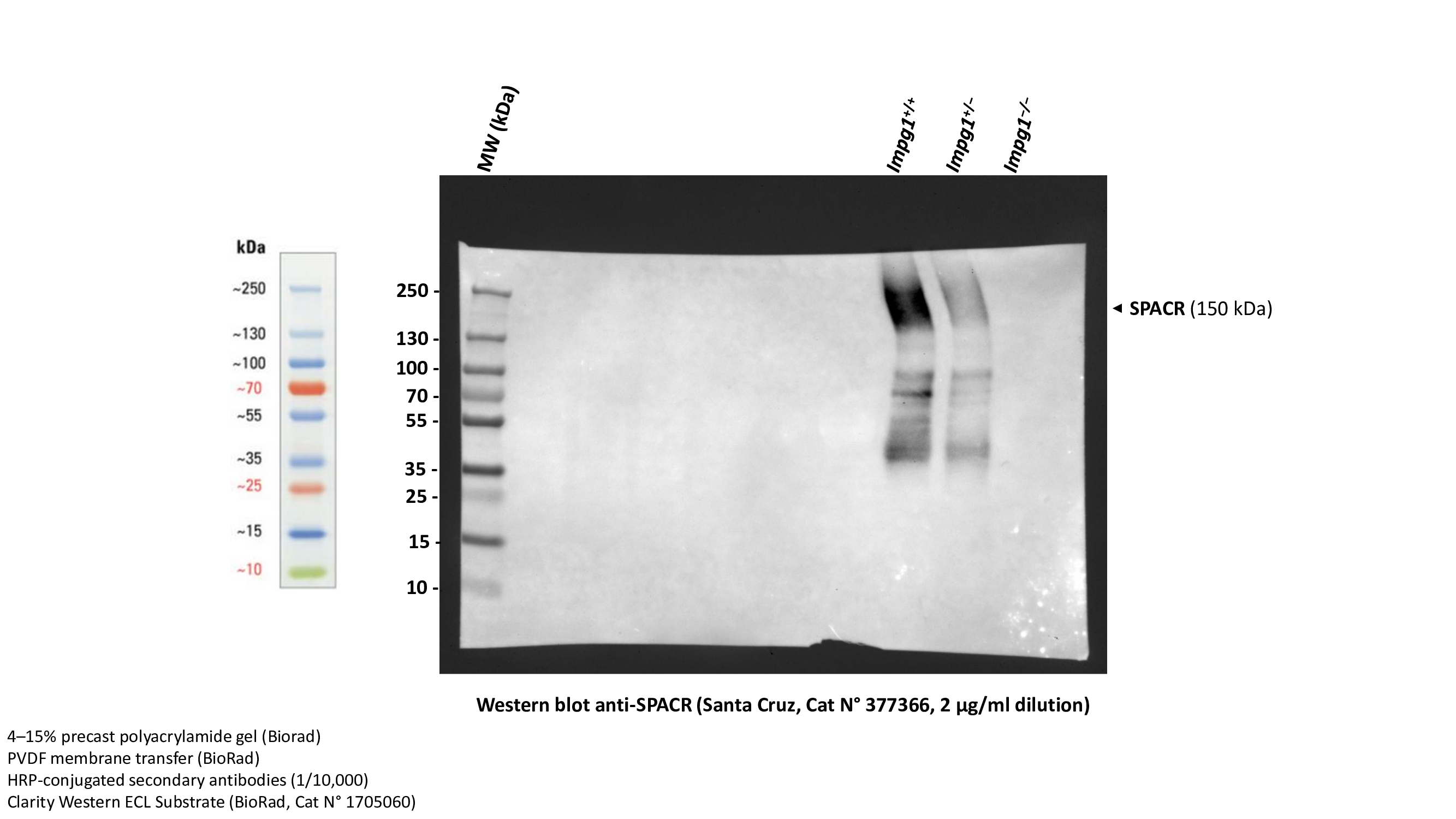

Supplement: Supplementary file 1 [file genes-13-01508-s001.zip › Supplementary_figure_S1.tiff]
